# Cloning, distribution, and effects of growth regulation of MC3R and MC4R in red crucian carp (*Carassius auratus* red var.)

**DOI:** 10.3389/fendo.2023.1310000

**Published:** 2024-01-23

**Authors:** Lu Huang, Xin Deng, Xiangqiong Yang, Zhong Tang, Siyu Fan, Zhifeng Zhou, Min Tao, Shaojun Liu

**Affiliations:** State Key Laboratory of Developmental Biology of Freshwater Fish, College of Life Sciences, Hunan Normal University, Changsha, Hunan, China

**Keywords:** red crucian carp, MC3R, MC4R, CRISPR/Cas9 system, energy homeostasis

## Abstract

**Background:**

Melanocortin-3 and -4 receptors (MC3R and MC4R), G protein-coupled receptors, play vital roles in the regulation of energy homeostasis. To understand the functions of *mc3r* and *mc4r* in the energy homeostasis of red crucian carp (*Carassius auratus* red var., RCC), we cloned *mc3r* and *mc4r*, analyzed the tissue expression and localization of the genes, and investigated the effects of knockout of *mc3r* (*mc3r* ^+/-^) and *mc4r* (*mc4r* ^+/-^) in RCC.

**Results:**

The full-length cDNAs of RCC *mc3r* and *mc4r* were 1459 base pairs (bp) and 1894 bp, respectively. qRT-PCR indicated that *mc3r* and *mc4r* were profusely expressed in the brain, but lower expressed in the periphery tissues. ISH revealed that *mc3r* and *mc4r* were located in NPP, NPO, NAPv, NSC, NAT, NRL, NLTl, and NLTp of the brain, suggesting that *mc3r* and *mc4r* might regulate many physiological and behavioral aspects in RCC. To further verify the roles of *mc3r* and *mc4r* in energy homeostasis, the *mc3r^+/-^* and *mc4r^+/-^* fish were obtained by the CRISPR/Cas9 system. The average body weights, total lengths, body depths, and food intake of *mc4r^+/-^* fish were significantly higher than those of *mc3r^+/-^* and the normal wild-type (WT) fish, but there was no difference between the *mc3r^+/-^* and WT fish, indicating that the RCC phenotype and food intake were mainly influenced by *mc4r* but not *mc3r*. Interestingly, *mc4r^+/-^* fish displayed more visceral fat mass than *mc3r^+/-^* and WT fish, and *mc3r^+/-^* fish also exhibited slightly more visceral fat mass compared to WT. RNA-seq of the liver and muscle revealed that a large number of differentially expressed genes (DEGs) differed in WT vs. *mc3r^+/-^*, WT vs. *mc4r^+/-^*, and *mc3r^+/-^* vs. *mc4r^+/-^*, mainly related to lipid, glucose, and energy metabolism. The KEGG enrichment analysis revealed that DEGs were mainly enriched in pathways such as steroid biosynthesis, fatty acid metabolism, fatty acid biosynthesis, glycolysis/gluconeogenesis, wnt signaling pathway, PPAR signaling pathway, and MAPK signaling pathway, thereby affecting lipid accumulation and growth.

**Conclusion:**

In conclusion, these results will assist in the further investigation of the molecular mechanisms in which MC3R and MC4R were involved in the regulation of energy homeostasis in fish.

## 1 Introduction

The melanocortins are post-translational products of proopiomelanocortin (POMC), consisting of α-, β-, and γ-melanocyte-stimulating hormones (MSHs) and adrenocorticotropic hormone (ACTH) that play important roles in multiple physiological functions via binding to melanocortin receptors (MCRs) (1, 2). MCRs, members of rhodopsin-like Family A G-protein-coupled receptors (GPCRs), comprise five members (MC1R to MC5R) with diverse physiological roles including skin and hair pigmentation, steroid secretion, energy homeostasis, and exocrine secretion (1).

MC3R and MC4R are mainly expressed in the central nervous system (CNS) and play critical roles in regulating energy homeostasis. To our knowledge, mutations of *mc3r* or *mc4r* in mice result in obesity (3, 4). The *mc4r* knockout mice show decreased energy expenditure, increased food consumption, increased fat mass, increased somatic growth, and substantial insulin resistance (3, 5). It is of note that the obesity phenotype of *mc3r* knockout mice are different from *mc4r* knockout mice, *mc3r* knockout mice do not exhibit significantly increased food intake, increased weight, and substantial insulin resistance, while they have increased fat mass and feed efficiency (4, 6). Recent data suggest that *mc3r* knockout mice are involved in triglyceride synthesis and hepatic lipogenesis (7). Likely, mutations of *mc3r* or *mc4r* in human are associated with obesity (5, 8, 9).

The relative expression of *mc3r* and *mc4r* has also been found in several peripheral tissues in addition to the central nervous system, indicating other potential physiological functions in the periphery with reproduction, cardiovascular, and immune response (1, 9–13). The MC3R and MC4R are primarily coupled to the stimulatory G protein (Gs) to stimulate the adenylyl cyclase activity, which will increase intracellular cAMP production to trigger downstream signaling (11, 14).

As in mammals, MC3R and MC4R have also attracted extensive attention in teleosts due to their functions in regulating energy homeostasis. The *mc3r* and *mc4r* are profusely expressed in CNS and widely expressed in peripheral tissues of topmouth culter (*Culter alburnus*) (9, 11). MC4R is involved in regulating the somatic growth, adipocyte hypertrophy, and energy homeostasis of zebrafish (*Danio rerio*) (15–17). In cavefish (*Astyanax mexicanus*), *mc4r* knockout fish have shown increased starvation resistance (18). In rainbow trout (*Oncorhynchus mykiss*), Schjolden et al. reported that MC4R could regulate the food intake in 2008 (19). The pharmacological properties of MC3R are investigated in channel catfish (*Ictalurus punctatus*) (8), common carp (*Cyprinus carpio*) (20), and zebrafish (21), indicating the functions in regulating energy homeostasis.

Red crucian carp (*Carassius auratus* red var.) is an ornamental fish with vivid red color and accounts for important proportion of freshwater aquaculture production worldwide. In this study, we investigated the physiological functions of MC3R and MC4R in red crucian carp by gene cloning, tissue distribution, and localization. To further verify the roles of MC3R and MC4R in regulating energy homeostasis and growth, we obtained the *mc3r* and *mc4r* knockout RCC and compared the difference of growth performance, lipid accumulation, DEGs, and KEGG pathways among different types. These findings laid the foundation for future physiological studies of teleosts MC3R and MC4R.

## 2 Materials and methods

### 2.1 Animal and ethics statement

All animal experiments were approved by the Animal Care Committee of Hunan Normal University and the Administration of Affairs Concerning Experimental Animals of China. Red crucian carp was collected from the Engineering Research Center of Polyploid Fish Reproduction and Breeding of the Ministry of Education at Hunan Normal University.

### 2.2 Gene cloning and sequence alignment

Red crucian carp was anesthetized before decapitation and the tissues were excised and stored at −80°C. Total RNA was purified by Trizol™ Reagent (Invitrogen, USA). The first-strand cDNA was synthesized by PrimeScript RT reagent Kit (TaKaRa, Japan). Primers, designed using Primer Premier 5.0 (**Supplementary Table 1**), were used to obtain full-length cDNA via PCR and touch-down PCR with TaKaRa LA Taq^®^. The PCR products were separated through 1.2% agarose gels, purified by SanPrep Column DNA Gel (Sangon, China), subcloned into the PMD18-T, and then sequenced (Sangon).

### 2.3 Quantitative reverse transcription PCR

To explore the tissue distribution of these genes, the medulla, mesencephalon, cerebellum, olfactory bulb, telencephalon, hypothalamus, pituitary, spleen, muscle, gonads (ovary and testis), gill, heart, liver, kidney, head kidney, and skin were obtained from three males (45.27 ± 4.79 g) and three females (46.33 ± 3.17 g), respectively. Primers were designed by AlleleID 6 and selected in qRT-PCR with 95%–105% amplification efficiency and single melting curve, and *β-actin* was used as the internal control (**Supplementary Table 1**). The qRT-PCR was carried out by a Prism 7,500 Sequence Detection System (ABI, USA). The 10-μL mixture of the reaction consisted of 5 μL of SYBR green PCR Master Mix (TaKaRa), 0.5 μL of each primer, 3 μL of ddH_2_O, and 1 μL of diluted cDNA sample. The qRT-PCR procedure was set as follows: 50°C for 2 min, 95°C for 10 min, followed by 40 cycles at 95°C for 15 s and 61°C for 45 s. The experimental samples were added to a 96-well plate repeated thrice. The relative expression of genes was calculated using the 2^−ΔΔCt^ method (22).

### 2.4 *In situ* hybridization

Primers (**Supplementary Table 1**) for the *in situ* hybridization (ISH) were designed by Primer Premier 5.0. Antisense digoxigenin (DIG)-labeled probes were synthesized through *in vitro* transcription using MAXIscript™ T7 *In Vitro* Transcription Kit (Invitrogen, USA) and purified by LiCl precipitation as described in the title (23). The probes were stored at −80°C.

The RCC brain was fixed in 4% paraformaldehyde (PFA, Sangon) for a night at 4°C. Next, the tissue was dehydrated using a series of graded sucrose solutions (15%, 20%, and 30%) and embedded in Tissue-Tek O.C.T. Compound^®^ (Sakura, USA). Then, the embedded tissues were cut using a freezing microtome (Leica CM3050 S Cryostat, Germany) into 20-μm-thick sections according to the instruction manual.

The ISH experiment needs to maintain an RNase-free environment. Prior to hybridization, the sections were washed with 1×PBST at room temperature (RT) for 5 min, treated with 5 μg/mL proteinase K at RT for 5 min, fixed with 4% PFA at RT for 10 min, and prehybridized (prehybridization solution was mixed as follows: 50 mL of deionized formamide, 25 mL of 20 × SSC, 920 μL of 1 M citric acid, 1 mL of 0.5 M EDTA, 500 μL of 20% Tween-20, 100 μL of 50 mg/mL heparin, 100 μL of 50 mg/mL tRNA, and 22.38 mL of DEPC-H2O) at 60°C for 4 h. Then, the sections were hybridized (hybridization solution was prepared by adding the RNA probe into prehybridization solution) at 60°C for 16 h. Post-hybridization, the sections were washed with 50% deionized formamide at 50°C for 15 min, 2×SSCT at RT for 15 min, and incubated with 2 μg/mL RNase A (Thermo Fisher Scientific, USA) buffer at RT for 30 min. Then, they were washed with 2×SSCT, 0.2×SSCT, and 1×PBST for 10 min, respectively. Next, the sections were blocked with blocking buffer at RT for 2 h and then incubated with AP-conjugated anti-DIG antibody (Roche, Germany, 1:3,000 diluted in the blocking buffer) overnight at 4°C. The sections were stained with NBT/BCIP stock solution (Roche, Germany) and the hybridization signals were detected using light microscopy (Olympus, Japan). The negative control was incubated with PBS instead of probes.

### 2.5 Gene editing via the CRISPR/Cas9 system

The CRISPR/Cas9 system was made up of Cas9 protein and guide RNA (gRNA) (24). gRNAs were designed and generated using the Maxiscript T7 PCR-based method. The gRNAs targeting *mc3r* and *mc4r* were designed using the online software tool (http://zifit.partners.org/ZiFiT) and two gRNAs of each gene were selected to ensure the knockout efficiency. Taking the DR274 plasmid as a template, the gRNAs were synthesized with corresponding primers (**Supplementary Table 1**) using Takara EX Taq (TaKaRa, Japan). The PCR products were confirmed with 1.2% agarose gel and used as templates in subsequent *in vitro* transcription reactions following the manufacturer’s guidelines of TranscriptAid T7 High Yield Transcription Kit (Thermo Fisher Scientific, USA) for synthesizing the gRNAs.

The TrueCut™ Cas9 Protein v2 was obtained from Thermo Fisher Scientific. The injection solutions were prepared by mixing Cas9 Protein and gRNAs, and then the mixture was incubated for 5 min on ice before being injected into the two-cell stage via a microinjector (PV830, WPI, USA). The final concentrations of gRNAs and Cas9 Protein were 200 ng/μL and 300 ng/μL, respectively.

Fin-clip samples of each fish were collected in sterile Eppendorf tubes and extracted genomic DNA using Tissue DNA Kit (Omega, USA) for mutation examination. The primers (**Supplementary Table 1**) for PCR amplification were encapsulated all possible mutation sites. Then, the amplified products were separated, purified, and sequenced as above.

### 2.6 Feeding trial

For this experiment, 6-month-old WT (*n* = 18, 10.28 ± 2.18 g), *mc3r* ^+/−^ (*n* = 18, 10.38 ± 2.27 g), and *mc4r* ^+/−^ (*n* = 18, 16.80 ± 4.68 g) fish were individually placed into nine tanks (six fish each) with the same feeding environment, and each type of fish was divided into three replicates. The fish were fed once a day. The feeding trial was carried out for 12 days and food intake was recorded daily. Food intake was calculated as the difference between the initial dry weight and residual dry weight.

### 2.7 Transcriptome sequencing

Total RNA was extracted from liver and muscle samples of treatment and control groups for transcriptome sequencing. The cDNA libraries were constructed using the NEBNext^®^ UltraTM RNA Library Prep Kit for Illumina^®^ (NEB, USA) according to manufacturer instructions. Then, the cDNA libraries were sequenced with Illumina HiSeq™ sequencing platform. The fastp v 0.19.3 was used to remove the low-quality bases and empty reads. Next, HISAT v2.1.0 was used to construct the index and compare clean reads to the reference genome (https://www.ncbi.nlm.nih.gov/nuccore/1764596402). The differential expressions of two groups were analyzed via DESeq2 v1.22.1. Genes with |log2Fold Change| ≥ 1 and false discovery rate (FDR) < 0.05 were defined as differentially expressed genes (DEGs). The DEGs enrichment analysis was performed based on the hypergeometric test. The Kyoto Encyclopedia of Genes and Genomes (KEGG) and gene ontology (GO) were performed based on the pathway and GO term, respectively.

### 2.8 Statistical analysis

DNAMAN version 5.0 was used to analyze multiple sequence alignment. Phylogenetic tree was constructed with MEGA 5 software. SPSS 19.0 software was used to calculate all data that were presented as the mean ± SEM. One-way ANOVA was used to analyze statistically significant differences in gene expression between multiple groups. The significance of differences in the average body weights, total lengths, body depths, food intake, visceral fat mass, and gene expression of mutated and control fish were determined by Student’s *t*-test. *p* < 0.05 was considered as statistically significant.

## 3 Results

### 3.1 Nucleotide and deduced amino acid sequences of RCC MC3R and MC4R

**Figure 1:**
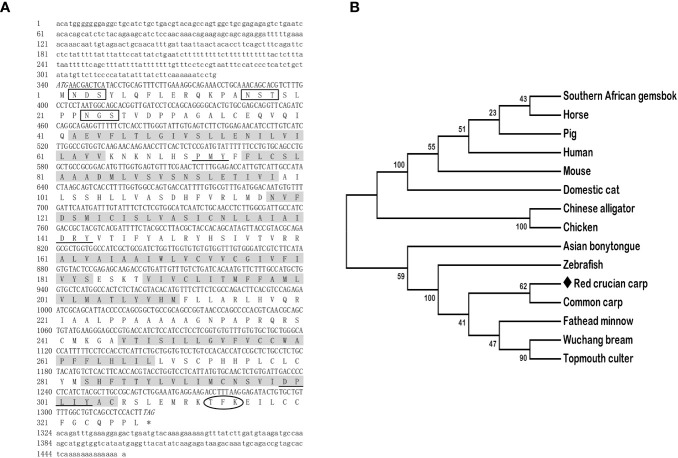
Nucleotide and deduced amino acid sequences and phylogenetic tree of RCC MC3R. **(A)** Nucleotide and deduced amino acid sequences of RCC MC3R. Lowercase letters and uppercase letters represent the non-coding regions and coding regions of the nucleotide sequence, respectively. Shaded boxes refer to putative TMD1-7. N-linked glycosylation sites are present in open boxes. Oval frame shows potential phosphorylation. Underlines denote PMY, DRY, and DPxxY motifs. Asterisk (*) shows stop codon. **(B)** Phylogenetic tree of RCC MC3R. The neighbor-joining (NJ) method was used to construct the phylogenetic tree. Numbers at nodes represent the bootstrap percentages, obtained for 1,000 replicates. *Carassius auratus* red var. (red crucian carp, OR573936), *Cyprinus carpio* (common carp, XP_042585810.1), *Megalobrama amblycephala* (wuchang bream, XP_048067317.1), *Danio rerio* (zebrafish, AAO24744.1), *Culter alburnus* (topmouth culter, QTW97901.1), *Pimephales promelas* (fathead minnow, XP_039535361.1), *Scleropages formosus* (Asian bonytongue, XP_018615783.1), *Alligator sinensis* (Chinese alligator, XP_006018246.1), *Oryx gazella* (southern African gemsbok, XP_040103238.1), *Mus musculus* (mouse, AAI03670.1), *Gallus gallus* (chicken, XP_040544507.1), *Sus scrofa* (pig, NP_001116609.1), *Equus caballus* (horse, NP_001243901.1), *Felis catus* (domestic cat, XP_023106851.2), and *Homo sapiens* (human, AKI72214.1).

**Figure 2:**
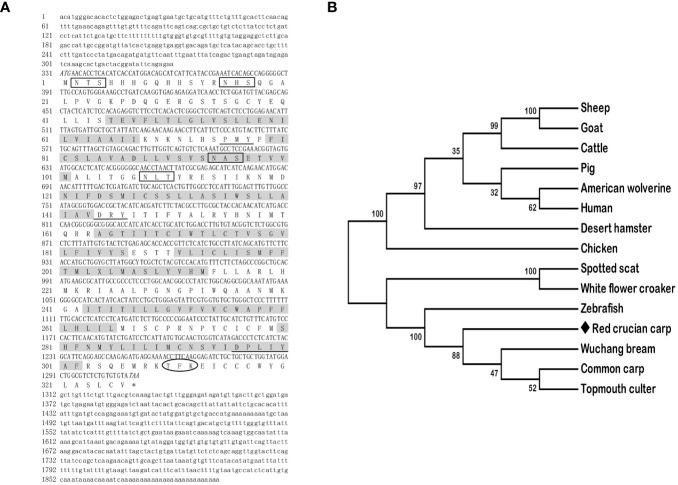
Nucleotide and deduced amino acid sequences and phylogenetic tree of RCC MC4R. **(A)** Nucleotide and deduced amino acid sequences of RCC MC4R. Lowercase letters and uppercase letters represent the non-coding regions and coding regions of the nucleotide sequence, respectively. Shaded boxes refer to putative TMD1-7. N-linked glycosylation sites are present in open boxes. Oval frame shows potential phosphorylation. Underlines denote PMY, DRY, and DPxxY motifs. Asterisk (*) shows stop codon. **(B)** Phylogenetic tree of RCC MC4R. The neighbor-joining (NJ) method was used to construct the phylogenetic tree. Numbers at nodes represent the bootstrap percentages, obtained for 1,000 replicates. *Carassius auratus* red var. (red crucian carp, OR573935), *Cyprinus carpio* (common carp, XP_042630234.1), *Megalobrama amblycephala* (wuchang bream, AWA81516.1), *Danio rerio* (zebrafish, NP_775385.1), *Culter alburnus* (topmouth culter, QKY77175.1), *Scatophagus argus* (spotted scat, AOQ25859.1), *Nibea albiflora* (white flower croaker, KAG8009637.1), *Phodopus roborovskii* (desert hamster, XP_051056768.1), *Ovis aries* (sheep, NP_001119842.1), *Capra hircus* (goat, NP_001272520.1), *Gulo gulo luscus* (American wolverine, KAI5773012.1), *Sus scrofa* (pig, NP_999338.1), *Bos taurus* (cattle, NP_776535.1), *Gallus gallus* (chicken, NP_001026685.2), and *Homo sapiens* (human, NP_005903.2).

The full-length cDNAs of *mc3r* (GenBank: OR573936) and *mc4r* (GenBank: OR573935) in red crucian carp were 1,459 base pairs (bp) and 1,894 bp, respectively. RCC *mc3r* included 339 bp 5′ UnTranslated Regions (UTR), 136 bp 3′UTR, and 984 bp open reading frame (ORF) encoding a putative protein of 327 amino acids (**Figure 1A**). RCC *mc4r* consisted of 331 bp 5′UTR, 582 bp 3′UTR, and 981 bp ORF encoding a putative protein of 326 amino acids (**Figure 2A**). Both RCC *mc3r* and *mc4r* had seven hydrophobic transmembrane domains (TMDs) and several conserved motifs (PMY, DRY, and DPxxY) that were significantly conserved with those of other species (**Figures 1** and **2**, and **Supplementary Figure 1**). Also, three potential N-linked glycosylation sites (Asn^2^, Asn^16^, and Asn^23^) were observed in RCC *mc3r* and four potential N-linked glycosylation sites (Asn^2^, Asn^15^, Asn^94^, and Asn^108^) were discovered in RCC *mc4r*. The consensus sequences for protein kinase C phosphorylation were found in *mc3r* (Thr^313^, Phe^314^, and Lys^315^) and *mc4r* (Thr^310^, Phe^311^, and Lys^312^), respectively (**Figures 1** and **2**). The results of multiple sequence alignment analysis showed that RCC MC3R and MC4R had higher positives with other piscinae and mammals (**Supplementary Figure 1**). Phylogenetic tree analysis revealed that RCC MC3R nested with common carp, fathead minnow, wuchang bream, and topmouth culter MC3Rs, and RCC MC4R nested with wuchang bream, common carp, topmouth culter, and zebrafish MC4Rs (**Figures 1B** and **2B**).

### 3.2 Tissue expression of RCC *mc3r* and *mc4r*

**Figure 3:**
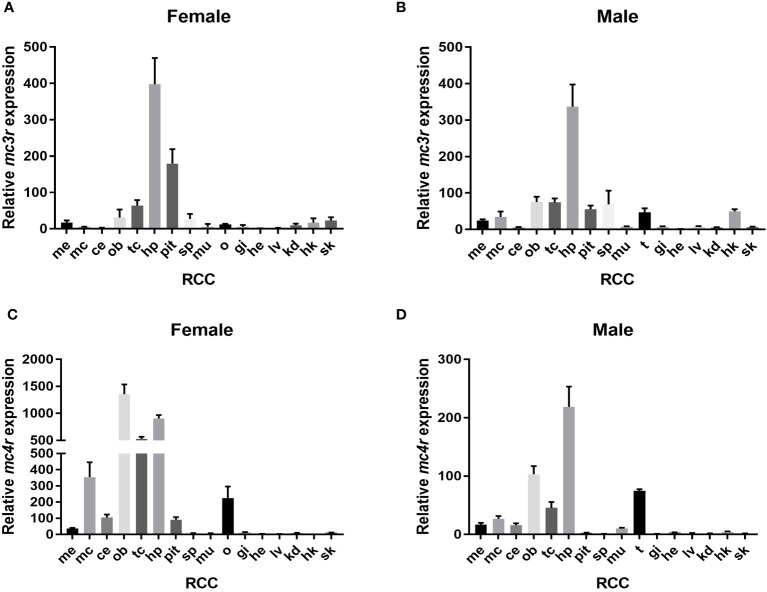
Relative expression of *mc3r* and *mc4r* in female and male RCC. **(A)** Relative expression of *mc3r* in female RCC. **(B)** Relative expression of *mc3r* in male RCC. **(C)** Relative expression of *mc4r* in female RCC. **(D)** Relative expression of *mc4r* in male RCC. The *β-actin* was used as the internal control. Data were shown as the mean ± SEM (*n* = 3). Me, medulla; mc, mesencephalon; ce, cerebellum; ob, olfactory bulb; tc, telencephalon; hp, hypothalamus; pit, pituitary gland; sp, spleen; mu, muscle; o, ovary; t, testis; gi, gill; he, heart; lv, liver; kd, kidney; hk, head kidney; sk, skin.

The qRT-PCR was used to analyze the relative expression of RCC *mc3r* and *mc4r*. The results reported that *mc3r* and *mc4r* were mainly expressed in the brain, and moderately expressed in peripheral tissues (**Figure 3**). In female RCC, *mc3r* was profusely expressed in the hypothalamus and pituitary gland but expressed at low levels in other tissues (**Figure 3A**). In male RCC, the highest expression of *mc3r* was observed in the hypothalamus, and also partially expressed in the olfactory bulb, telencephalon, spleen, testis, and head kidney (**Figure 3B**).

Similar to RCC *mc3r* expression. In female RCC, *mc4r* was highly expressed in the olfactory bulb, hypothalamus, telencephalon, mesencephalon, and ovary (**Figure 3C**). In male RCC, the relative expression of *mc4r* was higher in the hypothalamus, olfactory bulb, telencephalon, and followed by testis (**Figure 3D**).

### 3.3 The localization of RCC *mc3r* and *mc4r* in brain

ISH was used to further determine the localization of *mc3r* and *mc4r* in the RCC brain with antisense probes, with the PBS used as the negative control.

**Figure 4:**
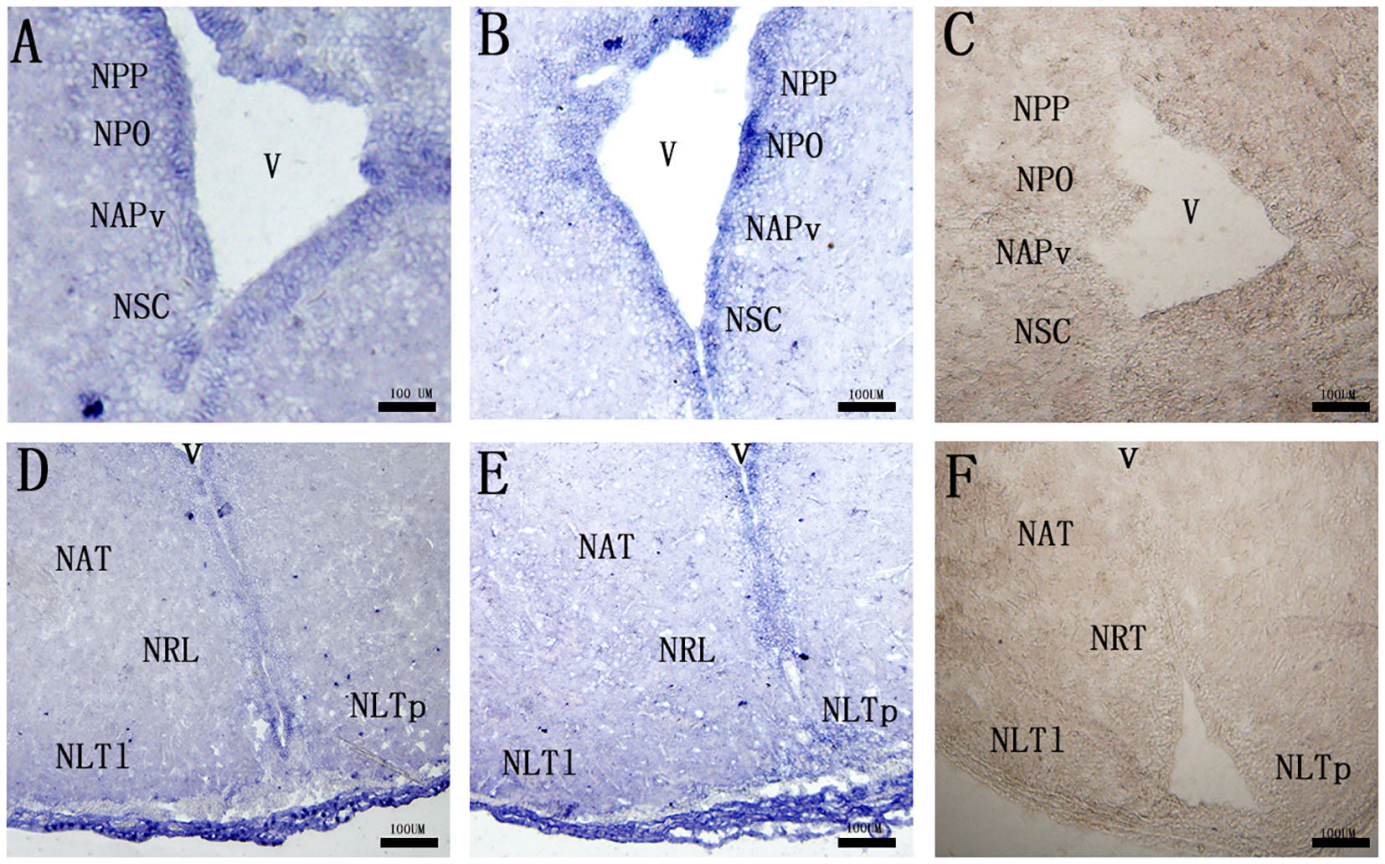
Localization of *mc3r* **(A, D)** and *mc4r* **(B, E)** in RCC brain. **(C, F)** represent negative control. The mRNA signals were determined by ISH. The periventricular part of the preoptic nucleus (NPP), magnocellular neurons of the preoptic nucleus (NPO), the anterior periventricular nucleus (NAPv), the suprachiasmatic nucleus (NSC), the anterior tyberal nucleus (NAT), the lateral recess nucleus (NRL), the posterior part of the lateral tuberal nucleus (NLTP), and the lateral part of the lateral tuberal nucleus (NLTl), third ventricle (v). Scale bar, 100 μm.

Both of the cell groups expressing *mc3r* and *mc4r* mRNAs were detected in several parts of the preoptic area and tuberal hypothalamus. In the preoptic area, *mc3r*-expressing and *mc4r*-expressing neurons were localized in the periventricular part of the preoptic nucleus (NPP), magnocellular neurons of the preoptic nucleus (NPO), the anterior periventricular nucleus (NAPv), and the suprachiasmatic nucleus (NSC), in which the expressions adopt a periventricular disposition (**Figures 4A, B**). Within the tuberal hypothalamus, positive *mc3r*-labeled and *mc4r*-labeled cells were evident in the anterior tuberal nucleus (NAT), the lateral recess nucleus (NRL), the posterior part of the lateral tuberal nucleus (NLTp), and the lateral part of the lateral tuberal nucleus (NLTl) (**Figures 4D, E**). No signals were found in the negative control (**Figures 4C, F**).

### 3.4 Generation of *mc3r* and *mc4r* mutations by the CRISPR/Cas9 system

**Figure 5:**
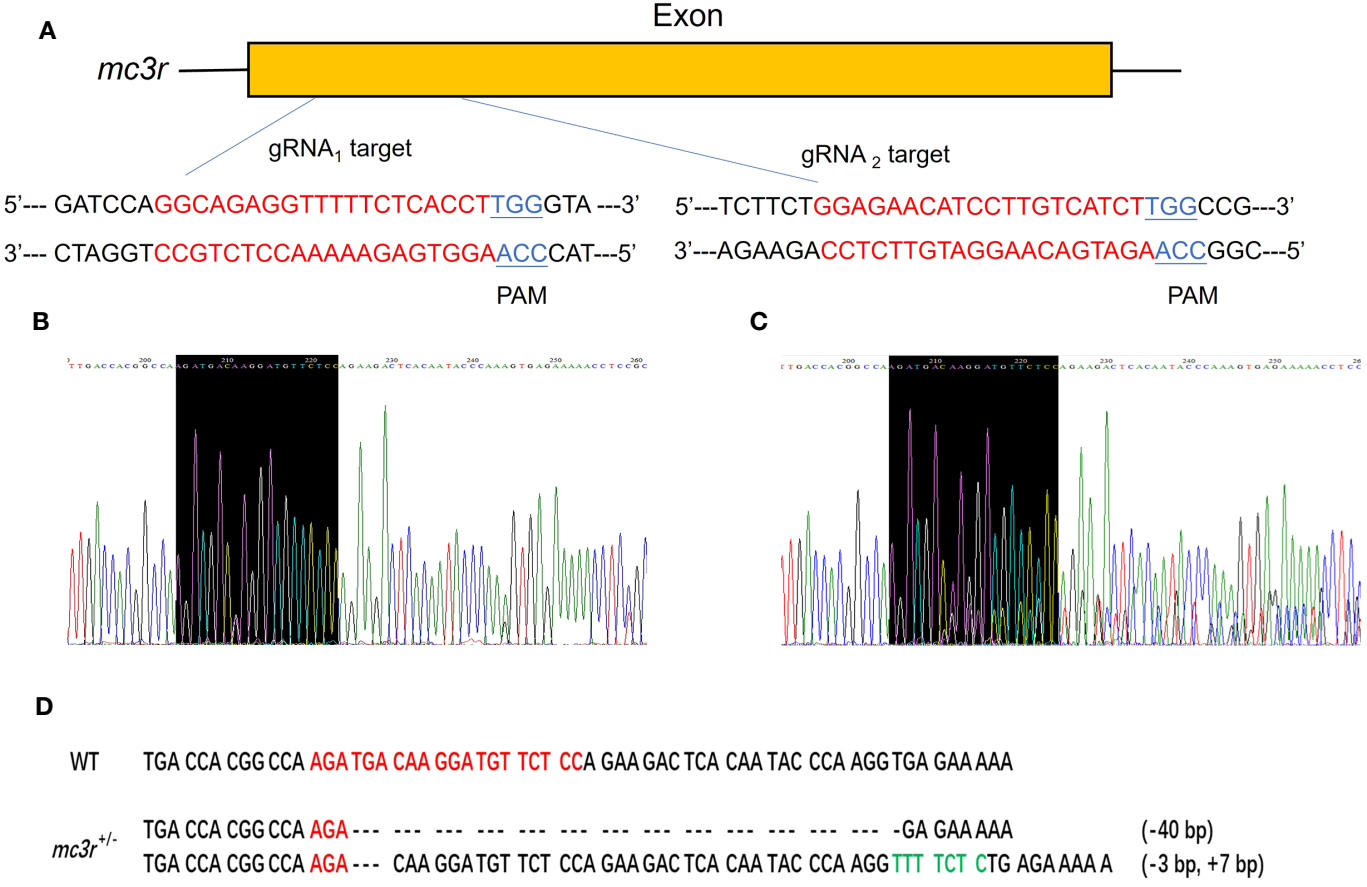
Generation of *mc3r*-deficient RCC with the CRISPR/Cas9 system. **(A)** Schematic representation of two genomic target sites on exon, respectively. The guide RNA target sites are indicated in red followed by PAM (Protospacer adjacent motif, NGG) in blue. **(B)** The Sanger sequencing result of WT. The black-shaded box denotes target site. **(C)** The Sanger sequencing result of *mc3r* ^+/−^ fish. The black-shaded box denotes target site. Multiple peaks occurred near the target sites. **(D)** DNA sequences of the mutants. Red sequences represent the guide RNA target sites, green sequences represent insertions, dashed lines represent deletions, and each dash corresponds to a nucleotide.

**Figure 6:**
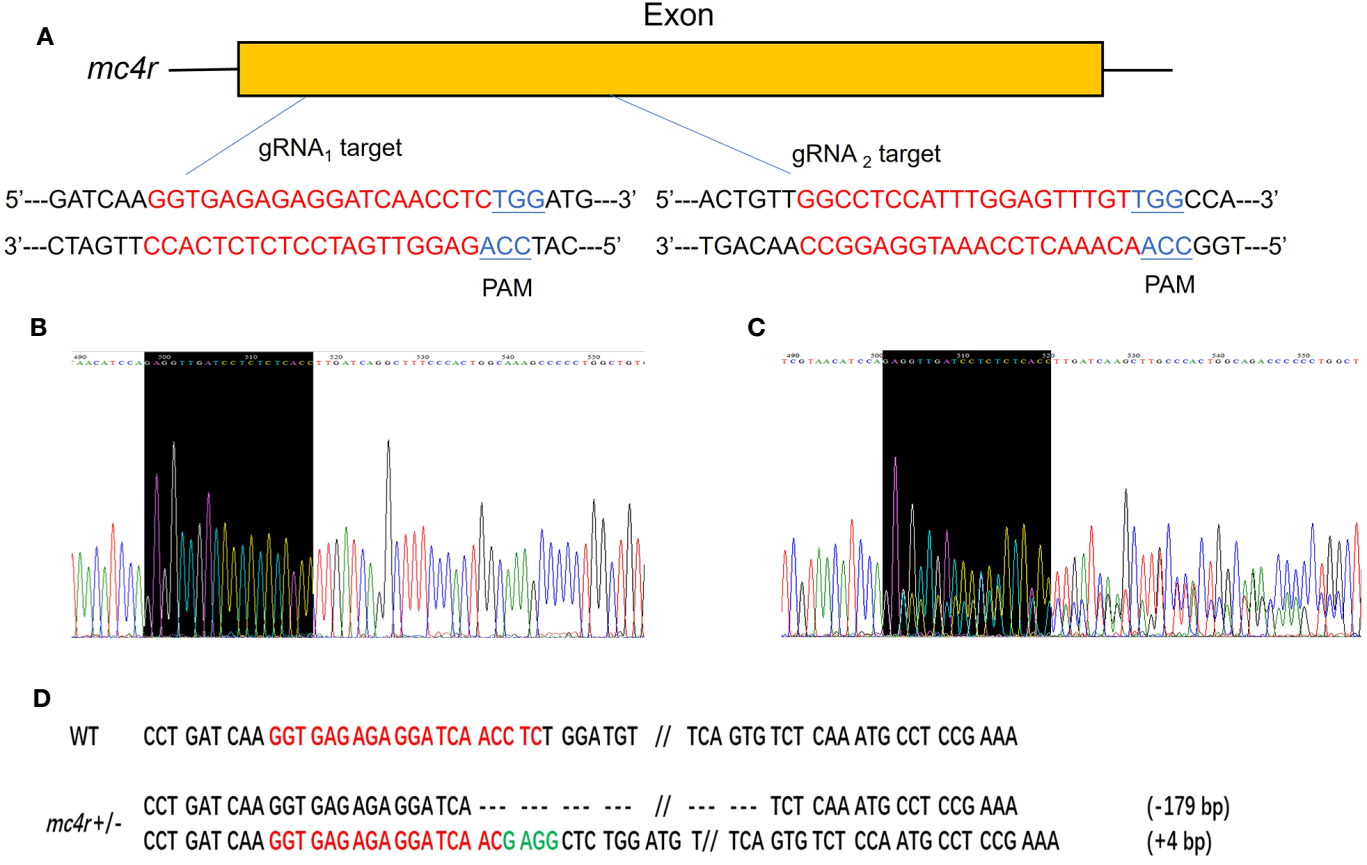
Generation of *mc4r*-deficient RCC with the CRISPR/Cas9 system. **(A)** Schematic representation of two genomic target sites on exon, respectively. The guide RNA target sites are indicated in red followed by PAM (Protospacer adjacent motif, NGG) in blue. **(B)** The Sanger sequencing result of WT. The black-shaded box denotes target site. **(C)** The Sanger sequencing result of *mc4r* ^+/−^ fish. The black-shaded box denotes target site. Multiple peaks are occurred near the target sites. **(D)** DNA sequences of the mutants. Red sequences represent the guide RNA target sites, green sequences represent insertions, double slash indicates that there is a large deletion, dashed lines represent deletions, and each dash corresponds to a nucleotide.

To verify the physiological functions of *mc3r* and *mc4r* in RCC energy homeostasis, the CRISPR/Cas9 system was used to mutate the RCC *mc3r* and *mc4r*. As we all know, genomic mutations contain either or all of the deletion, insertion, and substitution in the target areas at different levels. The PCR and Sanger sequencing revealed that multi-peaks were found in *mc3r*-gRNA_2_ and *mc4r*-gRNA_1_, the targeted areas (**Figures 5**, **6**). The gene cloning results of mutant *mc3r* and *mc4r* both had 2 different levels of mutations (**Figures 5D**, **6D**). There were two types of mutations in the mutant *mc3r*-gRNA_2_ group, including a type with 40-bp deletion, and another type with 3-bp deletion but 7-bp insertion (**Figure 5D**). Mutated fishes from the *mc4r*-gRNA_1_ group also exhibited two types of mutations, consisting of a type of 179-bp deletions and a type of 4-bp insertion (**Figure 6D**). The mutation rates were 32.3% in the *mc3r* mutation and 35.5% in the *mc4r* mutation. The survival rates of WT, *mc3r* ^+/−^, and *mc4r* ^+/−^ were 82.1%, 80.5%, and 57.7%, respectively.

### 3.5 Growth performance and food intake of the WT, *mc3r* ^+/−^, and *mc4r* ^+/−^

**Figure 7:**
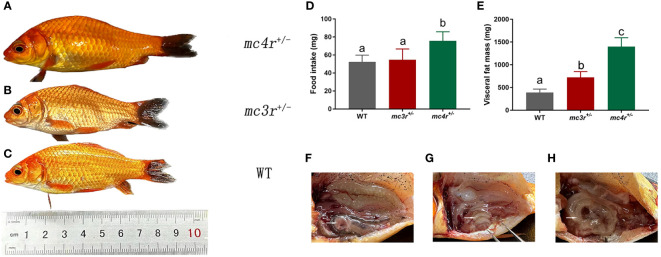
The growth, food intake, and visceral fat mass of WT and mutation fish. **(A)** The morphological observations of *mc4r* ^+/−^ fish. **(B)** The morphological observations of *mc3r* ^+/−^ fish. **(C)** The morphological observations of WT. **(D)** The food intake of WT, *mc3r* ^+/−^ and *mc4r* ^+/−^ fish. **(E)** The visceral fat mass of WT, *mc3r* ^+/−^, and *mc4r* ^+/−^ fish. **(F)** The anatomical diagram of WT. **(G)** The anatomical diagram of *mc3r* ^+/−^ fish. **(H)** The anatomical diagram of *mc4r* ^+/−^ fish. The white arrows in **(F–H)** point to the visceral adipose tissue. Different letters represent significant differences (*p* < 0.05). Data are shown as the mean ± SEM.

All individuals were fed in the tanks (height is 86 cm, diameter is 101 cm) with the same environment and fed twice a day at 9 a.m. and 5 p.m. The data of average body weights, total lengths, and body depths were measured at 6 months after hatching (mah) (**Table 1**; **Figures 7A–C**). The average body weights of WT, *mc3r* ^+/−^, and *mc4r* ^+/−^ fish were 10.91 ± 1.14 g (*n* = 8), 10.74 ± 0.42 g (*n* = 8), and 15.22 ± 2.45 g (*n* = 8), respectively. The average total lengths of WT, *mc3r* ^+/−^, and *mc4r* ^+/−^ fish were 8.70 ± 0.36 cm (*n* = 8), 8.72 ± 0.28 cm (*n* = 8), and 9.55 ± 0.34 cm (*n* = 8), respectively. The average body depths of WT, *mc3r* ^+/−^, and *mc4r* ^+/−^ fish were 2.75 ± 0.96 cm (*n* = 8), 2.78 ± 0.05 cm (*n* = 8), and 3.18 ± 0.22 cm (*n* = 8), respectively. The average body weights of *mc4r* ^+/−^ fish were 39.51% and 41.71% heavier than WT and *mc3r* ^+/−^ fish, respectively. The *mc4r* ^+/−^ fish had 9.77% and 9.52% increase in the average total lengths compared with WT and *mc3r* ^+/−^ fish, respectively. In addition, the rates of increase in the average body depths of *mc4r* ^+/−^ fish were 15.64% and 14.39% compared to WT and *mc3r* ^+/−^ fish, respectively. In a word, the average body weights, total lengths, and body depths of *mc4r* ^+/−^ fish were significantly higher than WT and *mc3r* ^+/−^ fish, but not between the WT and *mc3r* ^+/−^ fish. It is noted that the food intake of *mc4r* ^+/−^ fish was significantly increased compared to WT and *mc3r* ^+/−^ fish. However, there was no significant difference in food intake between WT and *mc3r* ^+/−^ fish (**Figure 7D**). In addition, it is more interesting that *mc4r* ^+/−^ fish displayed more visceral fat mass than WT and *mc3r* ^+/−^ fish. *mc3r* ^+/−^ fish also exhibited slightly more visceral fat mass compared to WT (**Figures 7E–H**). These data suggested that MC3R and MC4R play important roles in growth and lipid synthesis.

**Table 1:** Growth performance of WT, *mc3r* ^+/−^, and *mc4r* ^+/−^ RCC at 6 mah.

Group	Body weights (g)	Total lengths (cm)	Body depths (cm)
WT	10.91 ± 1.14^a^	8.70 ± 0.36 ^a^	2.75 ± 0.96 ^a^
*mc3r* ^+/−^	10.74 ± 0.42^a^	8.72 ± 0.28 ^a^	2.78 ± 0.05 ^a^
*mc4r* ^+/−^	15.22 ± 2.45^b^	9.55 ± 0.34 ^b^	3.18 ± 0.22 ^b^

Values with different superscripts in the same column are significantly different (p < 0.05). Data are shown as the mean ± SEM (n = 8). mah, months after hatching.

### 3.6 Transcriptome analysis and qRT-PCR verification

Transcriptome analysis of RCC WT, *mc3r* mutation, and *mc4r* mutation were performed to explain the mechanism of *mc3r* and *mc4r* in regulating energy homeostasis. The raw reads of liver and muscle were submitted to NCBI under accession PRJNA1018679 and PRJNA1018689, respectively.

#### 3.6.1 Analysis of DEGs

**Figure 8:**
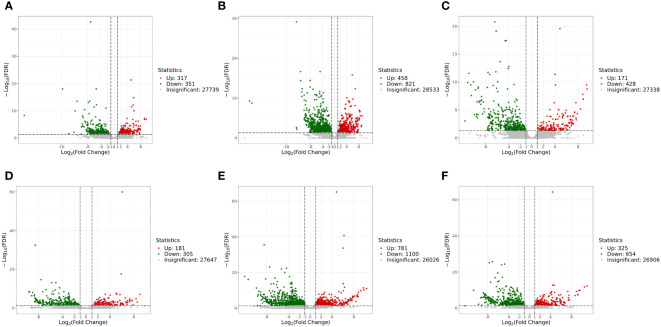
Volcano map of EDGs (*n* = 3). **(A)** Volcano map of EDGs between WT and *mc3r* ^+/−^ in liver. **(B)** Volcano map of EDGS between WT and *mc4r* ^+/−^ in liver. **(C)** Volcano map of EDGs between *mc3r* ^+/−^ and *mc4r* ^+/−^ in liver. **(D)** Volcano map of EDGs between WT and *mc3r* ^+/−^ in muscle. **(E)** Volcano map of EDGs between WT and *mc4r* ^+/−^ in muscle. **(F)** Volcano map of EDGs between *mc3r* ^+/−^ and *mc4r* ^+/−^ in muscle. Significantly up- and downregulated genes are highlighted in red and green, respectively, and insignificant genes are shown in gray.

**Figure 9:**
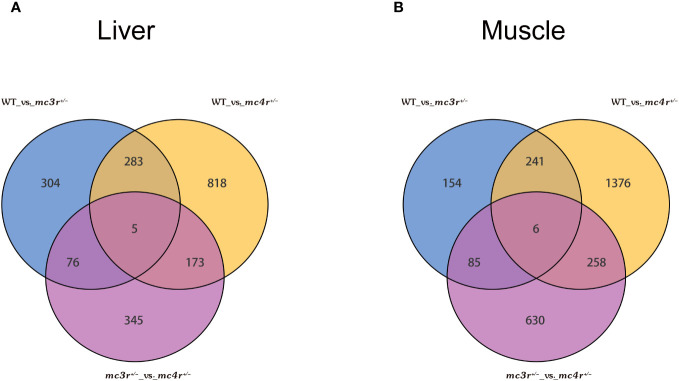
Venn diagram of DEGs (*n* = 3**). (A)** Venn diagram comparing the DEGs of WT vs. *mc3r* ^+/−^, WT vs. *mc4r* ^+/−^, and *mc3r* ^+/−^ vs. *mc4r* ^+/−^ in liver. **(B)** Venn diagram comparing the DEGs of WT vs. *mc3r* ^+/−^, WT vs. *mc4r* ^+/−^, and *mc3r* ^+/−^ vs. *mc4r* ^+/−^ in muscle. The non-overlapping region represents the unique DEGs for the differential group and the overlapping region represents the shared DEGs for the differential group.

The genes with |log2Fold Change| ≥ 1 and FDR < 0.05 were defined as DEGs. In the liver, there were 1,279 DEGs and 599 DEGs in the *mc4r* mutation group relative to the WT and *mc3r* mutation group, respectively, with 458 and 171 upregulated genes and the corresponding amounts of downregulated genes were 821 and 428. A total of 668 DEGs were identified in the *mc3r* mutation group compared to the WT group, including 317 upregulated genes and 351 downregulated genes (**Figures 8A–C**). As shown in the Venn diagram, the numbers of overlap DEGs in WT vs. *mc3r* ^+/−^ and WT vs. *mc4r* ^+/−^, WT vs. *mc3r* ^+/−^ and *mc3r* ^+/−^ vs. *mc4r* ^+/−^, and WT vs. *mc4r* ^+/−^ and *mc3r* ^+/−^ vs. *mc4r* ^+/−^ were 288, 81, and 178, respectively (**Figure 9A**).

In the muscle, the *mc4r* mutation group had 1,881 DEGs and 979 DEGs relative to the WT and *mc3r* mutation group, respectively, including 781 and 325 upregulated genes and 1,100 and 654 downregulated genes. The *mc3r* mutation group had 486 DEGs compared to the WT group, consisting of 181 upregulated genes and 305 downregulated genes (**Figures 8D–F**). As shown in the Venn diagram, the numbers of overlap DEGs in WT vs. *mc3r* ^+/−^ and WT vs. *mc4r* ^+/−^, WT vs. *mc3r* ^+/−^ and *mc3r* ^+/−^ vs. *mc4r* ^+/−^, and WT vs. *mc4r* ^+/−^ and *mc3r* ^+/−^ vs. *mc4r* ^+/−^ were 247, 91, and 264, respectively (**Figure 9B**).

#### 3.6.2 Go enrichment analysis of DEGs

**Figure 10:**
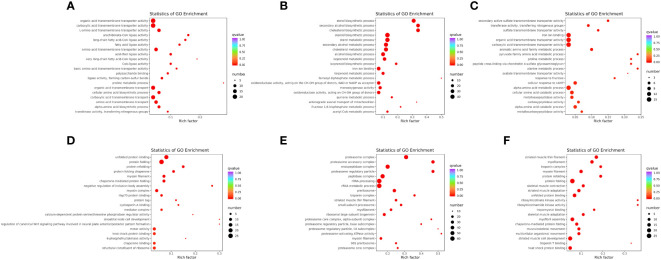
GO enrichment analysis of the DEGs in liver and muscle (*n* = 3). **(A)** GO enrichment analysis of the DEGs between WT and *mc3r* ^+/−^ in liver. **(B)** GO enrichment analysis of the DEGs between WT and *mc4r* ^+/−^ in liver. **(C)** GO enrichment analysis of the DEGs between *mc3r* ^+/−^ and *mc4r* ^+/−^ in liver. **(D)** GO enrichment analysis of the DEGs between WT and *mc3r* ^+/−^ in muscle. **(E)** GO enrichment analysis of the DEGs between WT and *mc4r* ^+/−^ in muscle. **(F)** GO enrichment analysis of the DEGs between *mc3r* ^+/−^ and *mc4r* ^+/−^ in muscle. The vertical coordinate represents the GO term. The horizontal coordinate represents the Rich factor. The larger the Rich factor, the greater the degree of enrichment. The larger the dot, the greater the number of DEGs enriched in the GO term. The redder the color of the dot, the more significant the enrichment.

GO annotation of DEGs was obtained by Blast2GO, and classified into three categories: biological process (BP), cellular component (CC), and molecular function (MF) (**Supplementary Figure 2**). Moreover, GO enrichment analysis of the WT vs. *mc3r* ^+/−^, WT vs. *mc4r* ^+/−^, and *mc3r* ^+/−^ vs. *mc4r* ^+/−^ in liver and muscle was performed, respectively, and the top 20 enrichment terms are shown in **Figure 10**.

In the liver, GO annotation of the three groups showed that most DEGs of the three groups were all annotated into BP and MF (**Supplementary Figures 2A–C**). GO enrichment analysis of WT vs. *mc3r* ^+/−^ showed that the enriched terms included organic acid transmembrane transporter activity, carboxylic acid transmembrane transporter activity, L-amino acid transmembrane transporter activity, arachidonate-CoA ligase activity, and long-chain fatty acid-CoA ligase activity (**Figure 10A**). In the WT vs. *mc4r* ^+/−^, the sterol biosynthetic process, secondary alcohol biosynthetic process, cholesterol biosynthetic process, steroid biosynthetic process, and sterol metabolic process were the most enriched (**Figure 10B**). In *mc3r* ^+/−^ vs. *mc4r* ^+/−^, secondary active sulfate transmembrane transporter activity, transferase activity, transferring nitrogenous groups, sulfate transmembrane transporter activity, iron ion binding, and organic acid transmembrane transporter activity were the most enriched (**Figure 10C**).

In the muscle, GO annotation of the three groups showed that most DEGs of the three groups were all annotated into BP (**Supplementary Figures 2D–F**). GO enrichment analysis of the WT vs. *mc3r* ^+/−^ showed that the enriched terms included unfolded protein binding, protein folding, protein refolding, protein folding chaperone, and myosin filament (**Figure 10D**). In the WT vs. *mc4r* ^+/−^, the enriched terms included proteasome complex, proteasome accessory complex, endopeptidase complex, proteasome regulatory particle, and peptidase complex (**Figure 10E**). In *mc3r* ^+/−^ vs. *mc4r* ^+/−^, striated muscle thin filament, myofilament, troponin complex, myosin filament, and protein refolding were the most enriched (**Figure 10F**).

#### 3.6.3 KEGG enrichment analysis of DEGs

**Figure 11:**
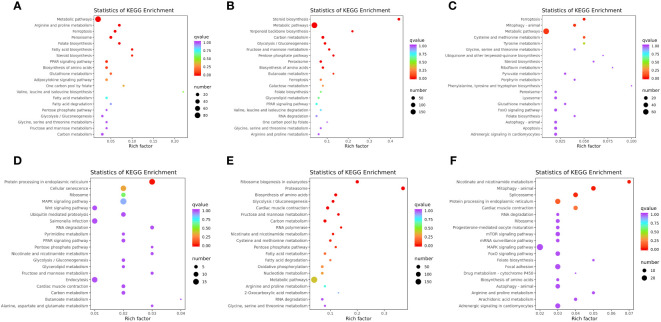
KEGG enrichment analysis of DEGs in liver and muscle (*n* = 3). **(A)** KEGG enrichment analysis of the DEGs between WT and *mc3r* ^+/−^ in liver. **(B)** KEGG enrichment analysis of the DEGs between WT and *mc4r* ^+/−^ in liver. **(C)** KEGG enrichment analysis of the DEGs between *mc3r* ^+/−^ and *mc4r* ^+/−^ in liver. **(D)** KEGG enrichment analysis of the DEGs between WT and *mc3r* ^+/−^ in muscle. **(E)** KEGG enrichment analysis of the DEGs between WT and *mc4r* ^+/−^ in muscle. **(F)** KEGG enrichment analysis of the DEGs between *mc3r* ^+/−^ and *mc4r* ^+/−^ in muscle. The vertical coordinate represents the GO term. The horizontal coordinate represents the Rich factor. The larger the Rich factor, the greater the degree of enrichment. The larger the dot, the greater the number of DEGs enriched in the GO term. The redder the color of the dot, the more significant the enrichment.

KEGG is a public pathway-related database, which is widely used to identify the biological pathways of DEGs. In the liver, a total of 122 pathways were enriched in WT vs. *mc3r* ^+/−^, 23 of which were significantly enriched (*p* < 0.05), such as metabolic pathways, arginine and proline metabolism, fatty acid biosynthesis, steroid biosynthesis, PPAR signaling pathway, adipocytokine signaling pathway, fatty acid metabolism, fatty acid degradation, and glycolysis/gluconeogenesis (**Figure 11A**). The representative genes related to lipid, glucose, and energy metabolism were *FACL4*, *fabp7a*, *LDHA*, *mthfr*, and *odc1*. In WT vs. *mc4r* ^+/−^, the DEGs were enriched in 149 pathways, and 34 pathways were significantly enriched (*p* < 0.05), including steroid biosynthesis, metabolic pathways, terpenoid backbone biosynthesis, carbon metabolism, glycolysis/gluconeogenesis, fructose and mannose metabolism, and PPAR signaling pathway (**Figure 11B**). The representative genes related to lipid, glucose, and energy metabolism were *sc5d*, *dhcr7*, *gamt*, *eno1*, *eno3*, and *acat2*. In *mc3r* ^+/−^ vs. *mc4r* ^+/−^, the DEGs were enriched in 117 pathways, and 21 pathways were significantly enriched (*p* < 0.05), such as metabolic pathways; cysteine and methionine metabolism; glycine, serine, and threonine metabolism; steroid biosynthesis; pyruvate metabolism; and FoxO signaling pathway (**Figure 11C**). The representative genes related to lipid, glucose, and energy metabolism were *pck1*, *foxo1a*, *gatm, acot12*, and *cdo1*.

In the muscle, a total of 101 pathways were enriched in WT vs. *mc3r* ^+/−^, 12 of which were significantly enriched (*p* < 0.05), such as MAPK signaling pathway, wnt signaling pathway, PPAR signaling pathway, glycolysis/gluconeogenesis, glycerolipid metabolism, and carbon metabolism (**Figure 11D**). The representative genes related to lipid, glucose, and energy metabolism were *DUSP8*, *irak4*, *gpam*, *mgll*, and *lipg*. In WT vs. *mc4r* ^+/−^, the DEGs were enriched in 152 pathways, and 31 pathways were significantly enriched (*p* < 0.05), such as the proteasome, biosynthesis of amino acids, glycolysis/gluconeogenesis, carbon metabolism, cysteine and methionine metabolism, fatty acid metabolism, and fatty acid degradation (**Figure 11E**). The representative genes related to lipid, glucose, and energy metabolism were *psma4*, *psma6*, *psmb4*, *acadl*, *cpt1*, *cpt2*, and *mat2b*. In *mc3r* ^+/−^ vs. *mc4r* ^+/−^, the DEGs were enriched in 128 pathways, and 16 pathways were significantly enriched (*p* < 0.05), such as nicotinate and nicotinamide metabolism, mTOR signaling pathway, MAPK signaling pathway, FoxO signaling pathway, biosynthesis of amino acids, and arginine and proline metabolism (**Figure 11F**). The representative genes related to lipid, glucose, and energy metabolism were *nampta*, *SGK1*, *irs1*, and *IGF*-*1R*.

#### 3.6.4 DEGs verification by qRT-PCR

**Figure 12:**
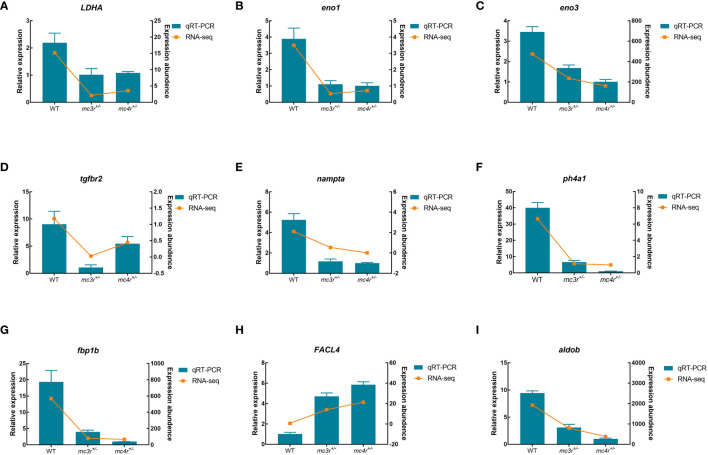
Results of qRT-PCR for nine genes of WT, *mc3r* ^+/−^, and *mc4r* ^+/−^ in liver. **(A)** *LDHA*, **(B)** *eno1*, **(C)** *eno3*, **(D)** *tgfbr2*, **(E)** *nampta*, **(F)** *p4ha1*, **(G)** *fbp1b*, **(H)** *FACL4*, and **(I)** *aldob*. The *β-actin* was used as the internal control. The data were shown as the mean ± SEM (*n* = 3) .

**Figure 13:**
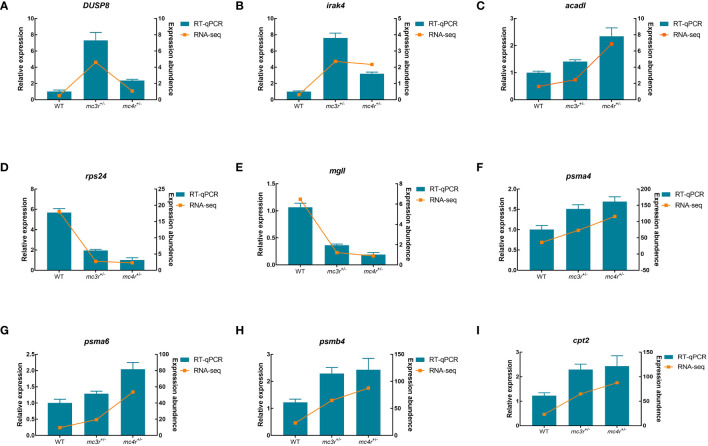
Results of qRT-PCR for nine genes of WT, *mc3r* ^+/−^, and *mc4r* ^+/−^ in muscle. **(A)** *DUSP8*, **(B)** *irak4*, **(C)** *acadl*, **(D)** *rps24*, **(E)** *mgll*, **(F)** *psma4*, **(G)** *psma6*, **(H)** *psmb4*, and **(I)** *cpt2*. The *β-actin* was used as the internal control. The data were shown as the mean ± SEM (*n* = 3).

To verify the dependability of transcriptome data, the DEGs associated to lipid metabolism, glucose metabolism, and amino acid metabolism were selected for qRT-PCR validation in the liver and muscle of the three groups. In the liver, *LDHA*, *eno1*, *eno3*, *tgfbr2*, *nampta*, *p4ha1*, *fbp1b*, *FACL4*, and *aldob* were used for qRT-PCR validation (**Figure 12**). In muscle, *DUSP8*, *irak4*, *acadl*, *rps24*, *mgll*, *psma4*, *psma6*, *psmb4*, and *cpt2* were used for qRT-PCR validation (**Figure 13**). The primers were designed based on transcriptome sequencing data (**Supplementary Table 1**). Our results showed that all the relative expression levels of the above genes had a similar trend with transcriptome data, indicating the dependability of transcriptome sequencing.

## 4 Discussion

In the present study, we cloned the full-length cDNAs of *mc3r* and *mc4r* in red crucian carp, which were 1,459 bp and 1,894 bp, respectively (**Figures 1A** and **2A**). The *mc3r* had a 984-bp ORF encoding a putative protein of 327 amino acids and the *mc4r* had a 981-bp ORF encoding a putative protein of 326 amino acids (**Figures 1A** and **2A**). Multiple sequence alignment revealed that RCC MC3R and MC4R both had seven TMDs and several conserved motifs as other species (**Figures 1A** and **2A**, and **Supplementary Figure 1**). Phylogenetic analysis showed that MC3R and MC4R clustered with teleost MC3Rs and MC4Rs, individually (**Figures 1B** and **2B**), consistent with previous studies (8, 9, 11, 25). These data indicated that RCC MC3R and MC4R shared high identity levels with MC3Rs and MC4Rs of other species, respectively.

We observed that RCC *mc3r* and *mc4r* were highly expressed in the brain, especially in the hypothalamus (**Figure 3**), indicating their roles in regulating energy homeostasis (9, 11, 25, 26). In addition, *mc3r* and *mc4r* were widely expressed in peripherical tissues, especially in the ovary and testis, indicating that they might play a vital function in regulating reproduction. Of note, several studies have confirmed the point that MC4R is associated with reproduction in various species (7, 13, 27–30). However, no data prove that MC3R is involved in regulating reproduction.

To further verify the functions of the central MC3R and MC4R in RCC, we detected the neuronal localization of *mc3r* and *mc4r* in the brain by ISH. In the brain, we found that the two receptor transcripts were observed in almost areas of the brain, such as the preoptic area, ventral thalamus, tuberal hypothalamus, and hypothalamic inferior lobe, and the positive signal of *mc4r* was stronger than *mc3r* (**Figure 4**). To our knowledge, the CNS is the center of appetite regulation, involved in regulating feeding, nutrient partitioning, and energy expenditure as in mammals (21, 31, 32). Previous reports have indicated that hormones involved in food intake such as NPY, AGRP, and were highly expressed in brain (33–35). Briefly, the *mc3r* and *mc4r* highly expressed in the brain might be associated with appetite regulation like the above hormones. We found that *mc4r* was extensively expressed in NPP, NPO, NAPv, NAT, NRL, NLTl, and NLTP (**Figures 4B, E**), consistent with the previous studies in rats and teleost (36–39). Evidence also shows that *mc4r* is expressed on NPY, GHRH, and AgRP neurons (40), and NPY could stimulate GH secretion (41). Similarly, *mc3r* was also detected in the same areas of the brain as the distribution of *mc4r* (**Figures 4A, D**). The distribution of *mc3r* is also observed in the hypothalamic arcuate nucleus of mice (42) and *mc3r* is expressed on POMC, GHSR, and NPY neurons, which are associated with feeding and growth (42, 43). In cyprinid fish, the NPP and NPO are primary corticotrophin-releasing hormone-producing areas (36, 44). The NLT of fish is regarded as a homologue of mammalian arcuate nucleus, which is a crucial receptor of feeding in mammals (40, 45). MC3R and MC4R co-expressed in these areas of the brain indicated that MC3R and MC4R might play crucial roles in regulating feeding and growth of RCC.

To further investigate the effects of MC3R and MC4R in growth, food intake, and energy homeostasis, the RCC *mc3r* ^+/−^ and *mc4r* ^+/−^ were generated by the CRISPR-Cas9 system with microinjection of gRNAs targeting the RCC *mc3r* and *mc4r* in conjunction with Cas9 protein. We found that the average body weights, total lengths, body depths, and food intake of *mc4r* ^+/−^ fish were significantly higher than WT and *mc3r* ^+/−^ fish, but there was no significant difference between WT and *mc3r* ^+/−^ fish at 6 mah (**Figures 7A–D**), indicating that RCC MC4R might play more important roles in growth and food intake than MC3R, consistent with previous studies (3–6, 19, 36). In addition, it is more interesting that RCC *mc4r* ^+/−^ displayed more visceral fat mass than *mc3r* ^+/−^ and WT. RCC *mc3r* ^+/−^ also exhibited slightly more visceral fat mass compared to WT (**Figures 7E–H**), indicating that MC3R and MC4R played an essential role in lipid accumulation and the role of MC4R was more significant than that of MC3R, consistent with previous studies in mice (3, 46). The *mc4r* mutants have been researched in several species including fish, pigs, and chickens (3, 47–52). In channel catfish, the body weights of *mc4r*-deficient fish exhibited 20%–38% improvement compared to WT (49). Holland’s carp containing SNP in the *mc4r* gene have more predominant growth traits than WT (52). Pigs and chickens containing SNPs in the *mc4r* gene also have more predominant growth traits than WT (48, 51). In addition, previous studies have proved that the body weights and fats of *mc3r*-deficient pigs were higher than WT (53). Chickens and blue foxes containing SNPs in the *mc3r* gene have more predominant growth traits than WT (54, 55). It can be seen that our data were consistent with those of previous studies. These data suggested that MC3R and MC4R were involved in growth regulation and food intake, but MC4R may play more important roles than MC3R. The improved growth in *mc3r*- and *mc4r*-deficient RCC might provide a new strategy for aquaculture.

The liver plays crucial roles in regulating glycogen storage, protein synthesis, and hormone production, and muscle is a major effector organ in fish (56, 57). Thus, we obtained and further analyzed the transcriptome sequences of liver and muscle in WT and *mc3r*- and *mc4r*-deficient RCC. Results show that a large of DEGs were found in the liver and muscle of WT vs. *mc3r* ^+/−^, WT vs. *mc4r* ^+/−^, and *mc3r* ^+/−^ vs. *mc4r* ^+/−^, and the DEGs mainly enriched in these pathways were related to growth, development, and energy metabolism (**Figures 8**, **9**, and **11**). In the liver, we found that *mc3r* deficiency could lead to the differential expression of the genes compared to WT like *FACL4*, *fabp7a*, *LDHA*, *mthfr*, and *odc1* in RCC. The *FACL4* plays an important role in lipid biosynthesis and fatty acid degradation (58). The *fabp* plays an essential role in intracellular lipid trafficking, and the upregulation of *fabp7a* can cause lipid accumulation (59–61). The *LDHA*-deficient mice exhibit moderately elevated body fat (62). The *mthfr* polymorphisms correlate with high homocysteine levels and subsequent insulin resistance and will cause overweight (63, 64). The *odc1*-deficient fruit fly exhibits increased number of fat cells and triglycerides (65). *mc4r* deficiency also led to the differential expression of the genes compared to WT like *sc5d*, *dhcr7*, *gamt*, *eno1*, *eno3*, and *acat2* in RCC. The *sc5d* and *gamt* play crucial roles in growth, lipid synthesis, and cellular metabolism (66, 67). The *dhcr7* is involved in steroid synthesis (68). The *acat2* is a key gene for fatty acid anabolism (69). The *eno1* has been found to be involved in the development, growth, and reproduction of organisms (70), and the *eno3* is well known for its functions in energy and lipid metabolism (71). In the *mc3r* ^+/−^ vs. *mc4r* ^+/−^, there were also some DEGs related to growth, lipid, and glucose metabolism like *pck1*, *foxo1a*, *gatm*, *acot12*, and *cdo1* in RCC. The *pck1* is a key gene for lipid metabolism and glucose homeostasis (72). The *foxo1a* is thought to be involved in controlling food intake (73). The *gatm* plays vital roles in lipid metabolism and energy homeostasis (67). The *acot12* and *cdo1* are suggested to be involved in regulating lipogenesis (74, 75).

In muscle, we found that *mc3r* deficiency could lead to the differential expression of the genes compared to WT like *DUSP8*, *irak4*, *gpam*, *mgll*, and *lipg* in RCC. The *DUSP8*-deficient mice can increase the energy expenditure and thus affect the body weight (76). The *irak4* can regulate the secretion of glucagon (77). The *gpam* is an enzyme in lipid metabolism and is thought to be influenced by the synthesis of triglyceride, cholesterol, and fatty acid contents in bovine mammary epithelial cells (78). The *mgll* is suggested to be involved in the regulation of lipid and biosynthesis and glycerolipid metabolism (79). The *lipg* is a member of the triglyceride lipase family and is integrally involved in lipid absorption, transport, and metabolism (80). *mc4r* deficiency also led to the differential expression of the genes compared to WT like *psma4*, *psma6*, *psmb4*, *acadl*, *cpt1*, *cpt2*, and *mat2b* in RCC. The *psma4* can regulate insulin sensitivity (81). The *psma6*, *mat2b*, and *psmb4* play essential roles in adipogenesis (82, 83). The *acadl*, *cpt1*, and *cpt2* are thought to be involved in lipolysis (84–86). In *mc3r* ^+/−^ vs. *mc4r* ^+/−^, there were also some DEGs related to growth, lipid, and glucose metabolism like *nampta*, *SGK1*, *irs1*, and *IGF*-*1R* in RCC. *nampta* is involved in regulating feeding and energy homeostasis in goldfish (87). *SGK1* may be associated with insulin secretion and obesity (88). The decreased expression of *irs1* may cause insulin resistance (89). The *IGF-1R* mutation has been suggested to affect skeletal development and growth in sheep (90). These data suggested that *mc3r* and *mc4r* mutations affected the differential expression of these genes involved in growth, lipid, and glucose metabolism, consistent with the above results regarding growth performance and lipid accumulation after *mc3r* and *mc4r* mutations in RCC.

In summary, we cloned the full-length cDNAs and analyzed the relative expression of *mc3r* and *mc4r* in RCC. Both genes were primarily present in the CNS, and widely present in the periphery. We also detected the localizations of *mc3r* and *mc4r* in the brain, and we found that the *mc3r*-expressing and *mc4r*-expressing neurons were localized in NPP, NPO, NAPv, NSC, NAT, NRL, NLTP, and NLTl, which were involved in regulating feeding and growth. The average body weights, total lengths, body depths, and food intake of *mc4r* ^+/−^ fish were significantly higher than those of *mc3r* ^+/−^ and WT fish, but there was no significant difference between *mc3r* ^+/−^ and WT fish. However, it is more interesting that RCC *mc4r* ^+/−^ displayed more visceral fat mass than *mc3r* ^+/−^ and WT. RCC *mc3r* ^+/−^ also exhibited slightly more visceral fat mass compared to WT. The data of RNA-seq showed that both *mc3r* and *mc4r* knockout affected the differential expression of many genes involved in growth, lipid, and glucose metabolism, indicating that *mc3r* and *mc4*r play vital roles in growth, lipid accumulation, lipidolysis, and insulin resistance. These findings laid the foundation for future physiological studies on the functions of *mc3r* and *mc4r* that might provide new strategies for improving the growth and aquaculture of teleost.

## Data availability statement

The datasets presented in this study can be found in online repositories. The names of the repository/repositories and accession number(s) can be found below: https://www.ncbi.nlm.nih.gov/bioproject/PRJNA1018679/, https://www.ncbi.nlm.nih.gov/bioproject/?term=PRJNA1018689, https://www.ncbi.nlm.nih.gov/nuccore/OR573936.1/, and Available at: https://www.ncbi.nlm.nih.gov/nuccore/OR573935. Accession numbers: PRJNA1018679, PRJNA1018679, OR573936, and OR573935.

## Ethics statement

The animal study was approved by Animal Care Committee of Hunan Normal University and the Administration of Affairs Concerning Experimental Animals of China. The study was conducted in accordance with the local legislation and institutional requirements.

## Author contributions

LH: Conceptualization, Data curation, Investigation, Writing – original draft. XD: Data curation, Writing – original draft. XY: Data curation, Writing – original draft. ZT: Data curation, Writing – original draft. SF: Data curation, Writing – original draft. ZZ: Data curation, Writing – original draft. MT: Conceptualization, Data curation, Funding acquisition, Project administration, Writing – review & editing. SL: Conceptualization, Data curation, Funding acquisition, Project administration, Writing – review & editing.

## Supplementary material

Supplementary Figure 1 Comparison of amino acid sequence between RCC and other species. **(A)** Comparison of amino acid sequence between RCC MC3R and MC3Rs from other species. **(B)** Comparison of amino acid sequence between RCC MC4R and MC4Rs from other species. Shaded boxes refer putative TMD1-7. Rectangular boxes denote PMY, DRY, DPxxY motifs. Carassius auratus red var. (red crucian carp, MC3R: OR573936), Cyprinus carpio (common carp, MC3R: XP_042585810.1), Megalobrama amblycephala (wuchang bream, MC3R: XP_048067317.1), Danio rerio (zebrafish, MC3R: AAO24744.1), Culter alburnus (topmouth culter, MC3R: QTW97901.1), Pimephales promelas (fathead minnow, MC3R: XP_039535361.1), Scleropages formosus (asian bonytongue, MC3R: XP_018615783.1), Alligator sinensis (chinese alligator, MC3R: XP_006018246.1), Oryx gazella (southern African gemsbok, MC3R: XP_040103238.1), Mus musculus (mouse, MC3R: AAI03670.1), Gallus gallus (chicken, MC3R: XP_040544507.1), Sus scrofa (pig, MC3R: NP_001116609.1), Equus caballus (horse, MC3R: NP_001243901.1), Felis catus (domestic cat, MC3R: XP_023106851.2), Homo sapiens (human, MC3R: AKI72214.1), Carassius auratus red var. (red crucian carp, MC4R: OR573935), Cyprinus carpio (common carp, MC4R: XP_042630234.1), Megalobrama amblycephala (wuchang bream, MC4R: AWA81516.1), Danio rerio (zebrafish, MC4R: NP_775385.1), Culter alburnus (topmouth culter, MC4R: QKY77175.1), Scatophagus argus (spotted scat, MC4R: AOQ25859.1), Nibea albiflora (white flower croaker, MC4R: KAG8009637.1), Phodopus roborovskii (desert hamster, MC4R: XP_051056768.1), Ovis aries (sheep, MC4R: NP_001119842.1), Capra hircus (goat, MC4R: NP_001272520.1), Gulo gulo luscus (american wolverine, MC4R: KAI5773012.1), Sus scrofa (pig, MC4R: NP_999338.1), Bos taurus (cattle, MC4R: NP_776535.1), Gallus gallus (chicken, MC4R: NP_001026685.2), Homo sapiens (human, MC4R: NP_005903.2).

Supplementary Figure 2 GO annotation analysis of DEGs in liver and muscle. **(A)** The result of WT vs. mc3r+/- in liver. **(B)** The result of WT vs. mc4r+/- in liver. **(C)** The result of mc3r+/- vs. mc4r+/- in liver. **(D)** The result of WT vs. mc3r+/- in muscle. **(E)** The result of WT vs. mc4r+/- in muscle. **(F)** The result of mc3r+/- vs. mc4r+/- in muscle.
