# Supplementary material for: Cloning, distribution, and effects of growth regulation of MC3R and MC4R in red crucian carp (Carassius auratus red var.)
Source: Front Endocrinol (Lausanne). 2024 Jan 23;14:1310000. doi: 10.3389/fendo.2023.1310000 (PMC10846643; doi:10.3389/fendo.2023.1310000)
Supplement: Supplementary file 1 [file Table_1.docx]

**Supplementary Table 1** Primers used in the present study.

| Primer names | Primer sequences (5′-3′) | Purpose |
| --- | --- | --- |
| *mc3r* -F | ATGGCAGCACGGTTGAT | PCR |
| *mc3r* -R | CAGAGGCAGAGCGGATG | PCR |
| *mc4r* -F | TCAAATGCCTCCGAAAC | PCR |
| *mc4r* -R  3′ sites adaptor  3′-*mc3r*-1  3′-*mc3r*-2  3′-*mc4r*-1  3′-*mc4r*-2  UPM (Mix)  NUP  5′-*mc3r*-1  5′-*mc3r*-2  5′-*mc4r*-1  5′-*mc4r*-2  *mc3r*-Q-F  *mc3r*-Q-R  *mc4r*-Q-F  *mc4r*-Q-R  *β-actin*-Q-F  *β-actin*-Q-R  *mc3r*-ISH-F  *mc3r*-ISH-R  *mc4r*-ISH-F  *mc4r*-ISH-R  *mc3r*-sgRNA_1_  *mc3r*-sgRNA_2_  *mc4r*-sgRNA_1_  *mc4r*-sgRNA_2_  Universal primer  *mc3r*-S-F  *mc3r*-S-R  *mc4r*-S-F  *mc4r*-S-R  *LDHA*-Q-F  *LDHA*-Q-R  *ENO1*-Q-F  *ENO1*-Q-R  *ENO3*-Q-F  *ENO3*-Q-R  *tgfbr2*-Q-F  *tgfbr2*-Q-R  *nampta* -Q-F  *nampta* -Q-R  *p4ha1* -Q-F  *p4ha1* -Q-R  *fbp1b* -Q-F  *fbp1b* -Q-R  *FACL4* -Q-F  *FACL4* -Q-R  *aldob* -Q-F  *aldob* -Q-R  *DUSP8* -Q-F  *DUSP8* -Q-R  *irak4* -Q-F  *irak4*-Q-R  *acadl* -Q-F  *acadl* -Q-R  *rps24* -Q-F  *rps24* -Q-R  *mgll* -Q-F  *mgll* -Q-R  *psma4* -Q-F  *psma4* -Q-R  *psma6* -Q-F  *psma6* -Q-R  *psmb4* -Q-F  *psmb4* -Q-R  *cpt1* -Q-F  *cpt1* -Q-R | ACGCCAGTCCATACCAG  CTGATCTAGAGGTACCGGATCC  TCTTCTCGCCAGACTTCACG  GACCATCTCCATCCTCCTCG  ATCCTGCTGGGAGTATTCGT  ATCTACGCATTCAGGAGCCA  CTAATACGACTCACTATAGGGCAAGCAGTGGTATCAACGCAGAGT (long)  AAGCAGTGGT AACAACGCAGAGT  GTGAGAAAAACCTCTGCCTGGAT  AAAAATACATCGGAGAGTGAAGGTT  AGGCGTAGAAGATCGTGATGTAG  CTACCGTTTCGGAGGCATTT  CGTGGTCTGTGGTATCGTCTTC  GCTGCTGCTGGAGGTAATGC  GCCTTATCAGCATGTTCTTCACC  CCAGCACACCACGAATACTCC  TCCCTTGCTCCTTCCACCA  GGAAGGGCCAGACTCATCGTA  GCAGCACGGTTGATCCTTCA  CAGTGAATTGTAATACGACTCACTATAGGGAGA  CTGTGGTAGCGTAAGGCGTA  GCCTCCGAAACGGTAGTGAT  CAGTGAATTGTAATACGACTCACTATAGGGAGA  CATGTGGACGTAGAGCGAGG  tgtaatacgactcactataGGCAGAGGTTTTTCTCACCTgttttagagctagaaatagc  tgtaatacgactcactataGGAGAACATCCTTGTCATCTgttttagagctagaaatagc  tgtaatacgactcactataGGTGAGAGAGGATCAACCTCgttttagagctagaaatagc  tgtaatacgactcactataGGCCTCCATTTGGAGTTTGTgttttagagctagaaatagc  AGCACCGACTCAGTGCCACT  CCTAATGGCAGCACGGTTGA  TCGCCAAGAGGTTGCAGATT  AGCACTGACTACGGATAT  TAGAGCGAAGCCATAAGC  CGGAGTCAATGTGGCTGGAG  ACCTCATACGCACTGTCAACC  CTACCGCCACATCGCAGAC  CCGCCGTTGATAACATTGAAGG  GCTGCTGAAGTCTGCCATCG  GCCGCTCCTGAAGAACTCG  GCTGTCGTCGTTCTCCTCTC  ATCGGTCGTCGTCGCTTAC  GTCTCCCATCAGCCCTCTCTC  GTGCTCTCATCCGCCGTTAG  AAGCATCGTAAACACAGCACAG  TCAACAGGAACCAACACAAAGC  GTTTGCTTTGACCCTCTGGATG  CCACAATCTCTCTGCCACTCC  TCTGCTTCTGCTGTCCTGTTG  GCTCCTACTCGTCCTGTGC  ATCAAGGACAAGGGCATCGTAG  CGCTCAGACAGACCATCCAATC  CGCTCGCTCATTCACATACG  CGCTTCTGACTCTGGCTCTG  CACCTGGAGCGACACTGAAG  GAGCCTCTGAACACGATGCC  GGTTGACAGCGGCACTAAGG  AGCAGGCAAACGCACATCC  ACGCCAAGATGAACGAGACC  CTGTTGCTTTGCCTGGATGC  GCAGGTGTGGTTCTCATAGGC  GATTGGGAGTCAAGCGGTTTAG  GTCCTCTGCCCTCGCTCTC  TGATTCTGGTCTTGCCGTTCTC  ACACACAGAACGCAGAGATGAG  CCTTGAAGCCGCAGTAATAACC  CGGGTAACAGCAGCAGTAGTG  CAGAGAGCCATAAGAGCCAAGC  ATGGTGAATGCCTATCCTCTGG  GGTTGCCTCGCCTTACTACG | PCR  3′ RACE  3′ RACE  3′ RACE  3′ RACE  3′ RACE  5′ RACE  5′ RACE  5′ RACE  5′ RACE  5′ RACE  5′ RACE  qRT-PCR  qRT-PCR  qRT-PCR  qRT-PCR  qRT-PCR  qRT-PCR  ISH  ISH  ISH  ISH  gRNA amplification  gRNA amplification  gRNA amplification  gRNA amplification  gRNA amplification  Sequencing and mutant screening  Sequencing and mutant screening  Sequencing and mutant screening  Sequencing and mutant screening  RNA-seq verification by qRT-PCR  RNA-seq verification by qRT-PCR  RNA-seq verification by qRT-PCR  RNA-seq verification by qRT-PCR  RNA-seq verification by qRT-PCR  RNA-seq verification by qRT-PCR  RNA-seq verification by qRT-PCR  RNA-seq verification by qRT-PCR  RNA-seq verification by qRT-PCR  RNA-seq verification by qRT-PCR  RNA-seq verification by qRT-PCR  RNA-seq verification by qRT-PCR  RNA-seq verification by qRT-PCR  RNA-seq verification by qRT-PCR  RNA-seq verification by qRT-PCR  RNA-seq verification by qRT-PCR  RNA-seq verification by qRT-PCR  RNA-seq verification by qRT-PCR  RNA-seq verification by qRT-PCR  RNA-seq verification by qRT-PCR  RNA-seq verification by qRT-PCR  RNA-seq verification by qRT-PCR  RNA-seq verification by qRT-PCR  RNA-seq verification by qRT-PCR  RNA-seq verification by qRT-PCR  RNA-seq verification by qRT-PCR  RNA-seq verification by qRT-PCR  RNA-seq verification by qRT-PCR  RNA-seq verification by qRT-PCR  RNA-seq verification by qRT-PCR  RNA-seq verification by qRT-PCR  RNA-seq verification by qRT-PCR  RNA-seq verification by qRT-PCR  RNA-seq verification by qRT-PCR  RNA-seq verification by qRT-PCR  RNA-seq verification by qRT-PCR |

F, Forward primer; R, Reverse primer.
