# Supplementary figures and images for: Cloning, distribution, and effects of growth regulation of MC3R and MC4R in red crucian carp (Carassius auratus red var.)
Source: Front Endocrinol (Lausanne). 2024 Jan 23;14:1310000. doi: 10.3389/fendo.2023.1310000 (PMC10846643; doi:10.3389/fendo.2023.1310000)

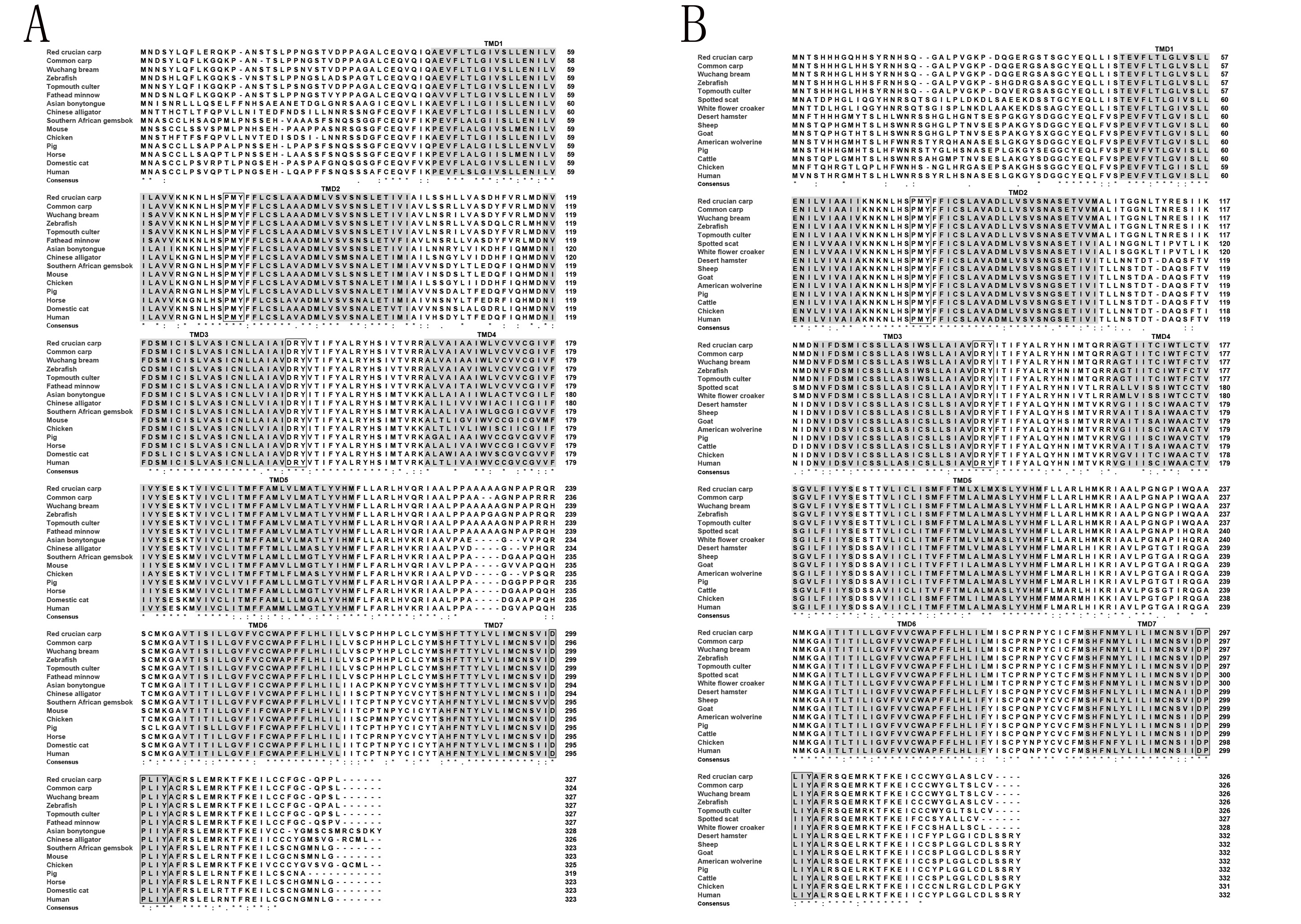

Supplement: Supplementary Figure 1 — Comparison of amino acid sequence between RCC and other species. (A) Comparison of amino acid sequence between RCC MC3R and MC3Rs from other species. (B) Comparison of amino acid sequence between RCC MC4R and MC4Rs from other species. Shaded boxes refer putative TMD1-7. Rectangular boxes denote PMY, DRY, DPxxY motifs. Carassius auratus red var. (red crucian carp, MC3R: OR573936), Cyprinus carpio (common carp, MC3R: XP_042585810.1), Megalobrama amblycephala (wuchang bream, MC3R: XP_048067317.1), Danio rerio (zebrafish, MC3R: AAO24744.1), Culter alburnus (topmouth culter, MC3R: QTW97901.1), Pimephales promelas (fathead minnow, MC3R: XP_039535361.1), Scleropages formosus (asian bonytongue, MC3R: XP_018615783.1), Alligator sinensis (chinese alligator, MC3R: XP_006018246.1), Oryx gazella (southern African gemsbok, MC3R: XP_040103238.1), Mus musculus (mouse, MC3R: AAI03670.1), Gallus gallus (chicken, MC3R: XP_040544507.1), Sus scrofa (pig, MC3R: NP_001116609.1), Equus caballus (horse, MC3R: NP_001243901.1), Felis catus (domestic cat, MC3R: XP_023106851.2), Homo sapiens (human, MC3R: AKI72214.1), Carassius auratus red var. (red crucian carp, MC4R: OR573935), Cyprinus carpio (common carp, MC4R: XP_042630234.1), Megalobrama amblycephala (wuchang bream, MC4R: AWA81516.1), Danio rerio (zebrafish, MC4R: NP_775385.1), Culter alburnus (topmouth culter, MC4R: QKY77175.1), Scatophagus argus (spotted scat, MC4R: AOQ25859.1), Nibea albiflora (white flower croaker, MC4R: KAG8009637.1), Phodopus roborovskii (desert hamster, MC4R: XP_051056768.1), Ovis aries (sheep, MC4R: NP_001119842.1), Capra hircus (goat, MC4R: NP_001272520.1), Gulo gulo luscus (american wolverine, MC4R: KAI5773012.1), Sus scrofa (pig, MC4R: NP_999338.1), Bos taurus (cattle, MC4R: NP_776535.1), Gallus gallus (chicken, MC4R: NP_001026685.2), Homo sapiens (human, MC4R: NP_005903.2). [file Image_1.jpeg]

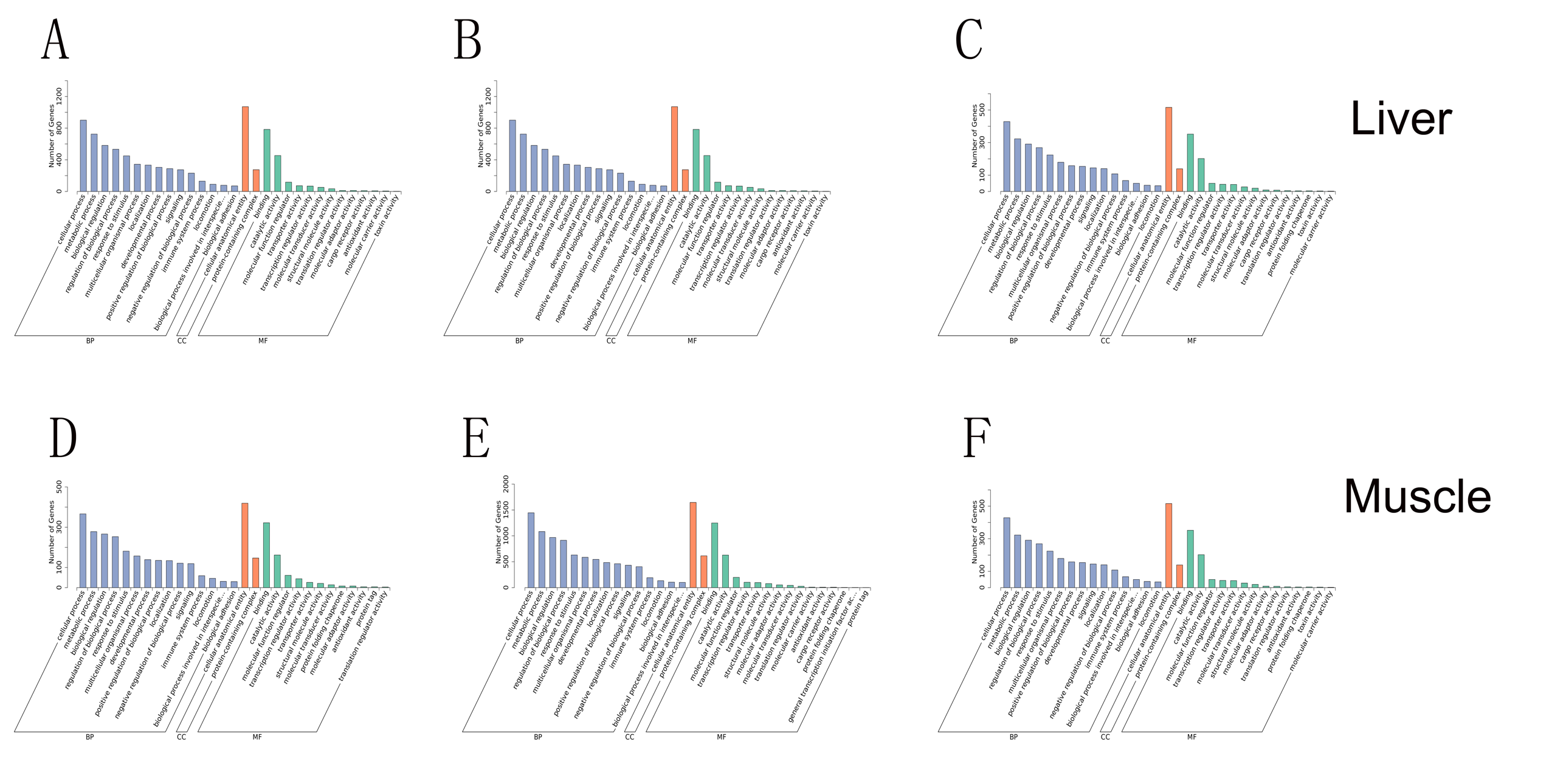

Supplement: Supplementary Figure 2 — GO annotation analysis of DEGs in liver and muscle. (A) The result of WT vs. mc3r+/- in liver. (B) The result of WT vs. mc4r+/- in liver. (C) The result of mc3r+/- vs. mc4r+/- in liver. (D) The result of WT vs. mc3r+/- in muscle. (E) The result of WT vs. mc4r+/- in muscle. (F) The result of mc3r+/- vs. mc4r+/- in muscle. [file Image_2.jpeg]
